# Fibrinogen Early In Severe Trauma studY (FEISTY): study protocol for a randomised controlled trial

**DOI:** 10.1186/s13063-017-1980-x

**Published:** 2017-05-26

**Authors:** James Winearls, Martin Wullschleger, Elizabeth Wake, Catherine Hurn, Jeremy Furyk, Glenn Ryan, Melita Trout, James Walsham, Anthony Holley, Jeremy Cohen, Megan Shuttleworth, Wayne Dyer, Gerben Keijzers, John F Fraser, Jeffrey Presneill, Don Campbell

**Affiliations:** 1 Gold Coast University Hospital, Southport, QLD Australia; 2 School of Medicine, University of Queensland, St. Lucia, QLD Australia; 3 School of Medical Sciences, Griffith University, Nathan, QLD Australia; 4 Royal Brisbane and Women’s Hospital, Herston, QLD Australia; 5 Emergency Research, Townsville Hospital, Douglas, QLD Australia; 6 Princess Alexandra Hospital, Woolloongabba, QLD Australia; 7 Townsville Hospital, Douglas, QLD Australia; 8 Intensive Care Research, Princess Alexandra Hospital, Woolloongabba, QLD Australia; 9 Menzies Health Institute Queensland, Griffith University, Nathan, QLD Australia; 10 Australian Red Cross Blood Service, Melbourne, VIC Australia; 11 School of Medicine, Bond University, Robina, QLD Australia; 12 Critical Care Research Group, The Prince Charles Hospital and University of Queensland, Brisbane, QLD Australia; 13 Intensive Care Unit, Royal Melbourne Hospital and University of Melbourne, Melbourne, VIC Australia

**Keywords:** Fibrinogen, Trauma, Haemorrhage, Cryoprecipitate, Fibrinogen concentrate, ROTEM®

## Abstract

**Background:**

Haemorrhage is a leading cause of death in severe trauma. Fibrinogen plays a critical role in maintaining haemostasis in traumatic haemorrhage. Early fibrinogen replacement is recommended by several international trauma guidelines using either fibrinogen concentrate (FC) or cryoprecipitate (Cryo). There is limited evidence to support one product over the other with widespread geographic and institutional variation in practice. This pilot trial is the first randomised controlled trial comparing FC to Cryo in traumatic haemorrhage.

**Methods/design:**

The Fibrinogen Early In Severe Trauma studY (FEISTY) is an exploratory, multicentre, randomised controlled trial comparing FC to Cryo for fibrinogen supplementation in traumatic haemorrhage. This trial will utilise thromboelastometry (ROTEM®) to guide and dose fibrinogen supplementation. The trial will recruit 100 trauma patients at four major trauma centres in Australia. Adult trauma patients with evidence of haemorrhage will be enrolled on arrival in the trauma unit and randomised to receiving fibrinogen supplementation with either FC or Cryo. The primary outcome is the differential time to fibrinogen supplementation. There are a number of predetermined secondary outcomes including: effects of the intervention on plasma fibrinogen levels, feasibility assessments and clinical outcomes including transfusion requirements and mortality.

**Discussion:**

The optimal method for replacing fibrinogen in traumatic haemorrhage is fiercely debated. In this trial the feasibility and efficacy of fibrinogen supplementation using FC will be compared to Cryo. The results of this pilot study will facilitate the design of a larger trial with sufficient power to address patient-centred outcomes.

**Trial registration:**

ClinicalTrials.gov, ID: NCT02745041. Registered 4 May 2016.

**Electronic supplementary material:**

The online version of this article (doi:10.1186/s13063-017-1980-x) contains supplementary material, which is available to authorized users.

## Background

Despite advances in trauma management over the last decade, haemorrhage in the setting of severe trauma remains a leading cause of morbidity and mortality [1, 2]. Death related to major haemorrhage is potentially preventable and represents a target for mortality reduction strategies. Traumatic haemorrhage is complicated by a complex coagulopathy – trauma-induced coagulopathy (TIC); the pathophysiological mechanisms of which are incompletely understood [3, 4].

Fibrinogen plays a critical role in maintaining effective haemostasis in traumatic haemorrhage and TIC [5, 6]. Fibrinogen is important in both primary haemostasis through platelet aggregation and secondary haemostasis where it is cleaved by thrombin to form fibrin [7, 8]. Fibrinogen is a glycoprotein synthesised in the liver and the healthy adult has a plasma fibrinogen concentration of between 2 to 4 g/L. Hypofibrinogenaemia and increased fibrinogen breakdown are key elements of TIC and fibrinogen is usually the first factor to reach critically low levels in traumatic haemorrhage [9, 10]. Hypofibrinogenaemia after severe trauma is associated with an increased risk of massive transfusion and death [11–13]. It is postulated that early fibrinogen replacement may assist in haemorrhage control, improve coagulopathy and reduce transfusion requirements [13–15]. Recently published trauma guidelines suggest that fibrinogen supplementation should occur in the presence of plasma fibrinogen levels <1.5–2 g/L or thromboelastometric signs of fibrinogen deficiency [16].

Major haemorrhage protocols (MHP) are a key component of the damage control approach to the severely bleeding trauma patient and the successful implementation of a MHP does improve outcomes [17, 18]. However, the ideal trauma MHP remains elusive, with considerable geographical and institutional variation [19, 20]. The three most commonly utilised MHP are: (1) high fixed-ratio blood product to packed red blood cell transfusion (PRBC) transfusion, (2) targeted blood product transfusion in response to viscoelastic haemostatic assay (VHA) results and (3) a hybrid approach utilising a high fixed ratio initially and subsequently transitioning to a VHA-guided approach [21, 22].

Regardless of which approach is used fibrinogen can be replaced with plasma, cryoprecipitate (Cryo) and fibrinogen concentrate (FC) each containing different amounts of fibrinogen; 2 g/L, 8–16 g/L and 20 g/L, respectively. The low concentration of fibrinogen in plasma and the large volumes required potentially make it unsuitable for dedicated fibrinogen supplementation [23–25]. The RETIC trial (NCT01545635) comparing plasma to factor concentrate-based resuscitation has recently been terminated early after an interim analysis revealed potential harm to patients randomised to the plasma arm. Cryoprecipitate contains substantially higher concentrations of fibrinogen than plasma and is widely accepted as the standard of care for fibrinogen supplementation in severe haemorrhage [26]. However, its use is not currently supported by high-level evidence [27]. The CRYOSTAT trial reported in 2015 that early fibrinogen supplementation utilising Cryo was feasible and effectively maintained fibrinogen levels in traumatic haemorrhage [28]. The use of FC has a number of theoretical advantages including: standard dose per vial, reduced volume, viral inactivation, no requirement for ABO compatibility matching, ease of reconstitution and administration. However, in severe trauma there are no robust clinical trials to demonstrate a survival or cost-effectiveness benefit compared to Cryo [6, 25, 29].

A number of key questions regarding fibrinogen supplementation in traumatic haemorrhage remain unanswered, including: (1) What tests, if any should be utilised to guide fibrinogen therapy? (2) What is the optimal product for fibrinogen replacement? (3) What is the optimal dose of fibrinogen? and (4) What is the impact of early fibrinogen replacement on clinically important outcomes?

The objectives of this trial are to assess the feasibility of early fibrinogen replacement utilising FC compared to the use of Cryo in patients with traumatic haemorrhage and viscoelastic evidence of hypofibrinogenaemia. We hypothesise that fibrinogen replacement to reverse hypofibrinogenaemia in traumatic haemorrhage may be achieved more rapidly, and with a more predictable dose response, using FC compared to Cryo.

## Methods/design

This is an investigator-initiated, pilot, multicentre, randomised controlled trial (RCT) comparing FC to Cryo for fibrinogen replacement in traumatic haemorrhage. The trial will take place in four major trauma centres in Queensland, Australia. The protocol adheres to the Standard Protocol Items: Recommendations for Interventional Trials (SPIRIT) Statement (Additional file 1).

### Patients

The trial will enrol 100 adult (aged 18 years or older) trauma patients judged by treating clinicians to have clinically significant haemorrhage or potential for significant transfusion requirements with an Assessment of Blood Consumption Score (ABC Score) ≥2. The ABC Score is a well-validated tool for the prediction of massive transfusion, has been utilised in a recent civilian trauma haemorrhage RCT and is simple to apply in the complex multidisciplinary environment of major trauma resuscitation [30–32]. See Table 1 for full inclusion and exclusion criteria.

**Table 1:** Inclusion and exclusion criteria

Inclusion criteria:	
Adult (≥18 years) affected by trauma *and*	
Judged to have significant haemorrhage *or*	
Predicted to require significant transfusion with ABC Score ≥2	
or by treating clinician judgment	
Exclusion criteria:	
Injury judged incompatible with survival	
Previous fibrinogen administration this admission	
Pretrauma centre dedicated fibrinogen replacement	
Known objection to blood products	
Known coagulation disorder	
Pregnancy	
Enrolment in competing study	

### Randomisation

A computer-generated 1:1 block randomisation schedule has been produced by a statistician independent to the study, accommodating unequal recruitment across the four initial sites together with the possibility of additional trial sites. Secure, password-protected web-based randomisation will occur on patient arrival to a participating hospital trauma unit once inclusion and exclusion criteria have been assessed. Each patient will receive fibrinogen supplementation comprising either protocol-directed FC or Cryo therapy. Allocation concealment will be maintained through to the conclusion of the web randomisation process, but for practical and safety reasons, randomised trial patients and their treating health care professionals will not be blind to the trial intervention. Trial integrity will be supported by maintenance of blinding for outcome assessors and the trial statistician.

### Intervention

**Fig. 1:**
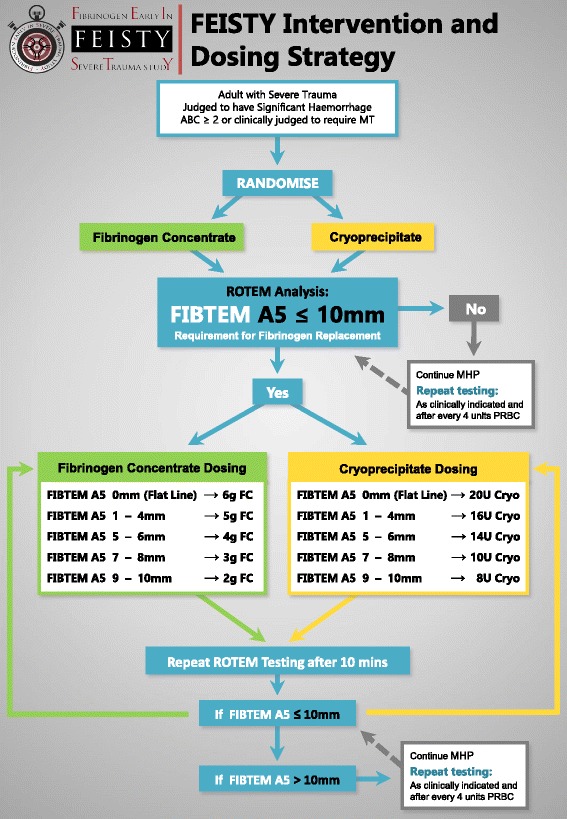
Intervention and dosing strategy

After compliance with consent requirements and with the treating clinician’s approval, patients will be randomised to receive fibrinogen supplementation using either FC (Riastap®, CSL Behring GmbH, Marburg, Germany) or Cryo (Australian Red Cross Blood Service). The requirement for fibrinogen supplementation will be defined by a FIBTEM A5 (clot amplitude 5 min after clot formation) ≤10 mm together with a clinical scenario suggesting significant haemorrhage. The intervention will be performed in randomised patients as soon as possible after each FIBTEM threshold result becomes known. Patients will remain in their allocated intervention arm for the duration of their hospital admission up to 30 days. Dosing of FC and Cryo will be guided by the FIBTEM A5 result as per trial protocol (Fig. 1). FC will be administered by intravenous injection and Cryo by rapid intravenous infusion. Fibrinogen supplementation will be continued throughout the resuscitation according to protocol and as per the allocated intervention arm (Fig. 1).

All other care received by trial patients will be prescribed by the managing staff according to relevant local hospital clinical and trauma guidelines that are based on Emergency Management of Severe Trauma (EMST) or Advanced Trauma Life Support (ATLS) principles. In all centres a Damage Control Resuscitation (DCR) approach to the management of severe trauma is standard practice; with rapid identification and treatment of life-threatening injuries, organ support and subsequent definitive injury management once the patient has been stabilised [33]. In patients with suspected haemorrhage the focus is geared towards rapid identification and surgical or interventional radiological control of haemorrhage. In all centres focused bedside ultrasound scan (USS) and whole body ‘Trauma’ computed tomography (CT) imaging when haemodynamic stability permits is standard of care. All centres have a ‘Red Blanket’ protocol with rapid transfer directly to the operating room (OR) for the haemodynamically unstable and exsanguinating trauma patient. Concurrent with haemorrhage control – haemostatic resuscitation (HR) is employed, involving blood product transfusion, correction of acidaemia, temperature control and organ support in an attempt to maintain ‘normal’ physiology.

### Trial endpoints

The primary outcome is the time interval in minutes from FIBTEM analysis and clinical scenario suggesting that fibrinogen supplementation is required to the completion of the first administration of fibrinogen supplementation (FC or Cryo). It is aimed to administer the FC intervention within 30 min of fibrinogen requirement being identified. Consistent with the exploratory pilot nature of this study, multiple hypothesis-generating outcomes will be evaluated together with assessments of process feasibility, including:

- Effects of fibrinogen supplementation (FC and Cryo) on plasma fibrinogen
- Time to randomisation
- Time course of fibrinogen levels as measured by FIBTEM and Clauss Fibrinogen (FibC)
- Transfusion requirements
- Clinically relevant thromboembolic complications
- Mortality at several time points including hospital discharge and 90 days

**Fig. 2:**
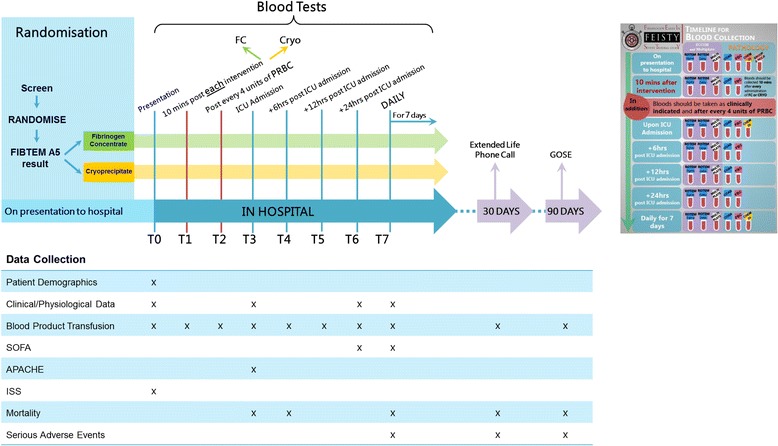
Fibrinogen Early In Severe Trauma studY (FEISTY) Standard Protocol Items: Recommendations for Interventional Trials (SPIRIT) flowchart

Detailed data collection will occur for the first 7 days of hospital admission and extended follow-up to 90 days (see the SPIRIT figure in Fig. 2). See Table 2 for full primary, secondary and feasibility outcomes.

**Table 2:** Outcome measures

Primary outcome measures:	
1. (A) Time to administration of fibrinogen administration (FC or Cryo) from time of ROTEM® analysis and clinical scenario suggesting that fibrinogen supplementation is required	
1. (B) Feasibility of administering FC within 30 min of ROTEM® and clinical scenario suggesting that fibrinogen supplementation is required	
2. Effects of fibrinogen supplementation (FC and Cryo) on fibrinogen levels as measured by FIBTEM and Clauss Fibrinogen (FibC)	
Secondary outcome measures:	
1. Transfusion requirements (in number of units) of PRBC, FFP, FC, Cryo, platelets, PCC at 4, 6, 12, 24 and 48 h	
2. Duration of bleeding episode or time until surgical haemorrhage control with no further coagulation factors	
3. Duration of mechanical ventilation	
4. Duration of ICU and hospital LOS	
5. ROTEM® (Sigma and Delta), Multiplate®, FBC, INR, APTT, FibC analysis at prespecified time points	
6. Evaluation of EXTEM CT in response to fibrinogen supplementation	
7. Adverse events: TACO, TRALI, Sepsis, MOF	
8. Thromboembolic complications	
9. All-cause mortality at 4, 6, 24 h and up to 90 days	
Feasibility outcome measures:	
1. Transfusion requirements (in number of units) of PRBC, FFP, FC, Cryo, Platelets, PCC at 4, 6, 12, 24 and 48 h	
2. Duration of bleeding episode or time until surgical haemorrhage control with no further coagulation factors	
3. Duration of mechanical ventilation	
4. Duration of ICU and hospital LOS	
5. ROTEM® (Sigma and Delta), Multiplate®, FBC, INR, APTT, FibC analysis at prespecified time points	
6. Evaluation of EXTEM CT in response to fibrinogen supplementation	
7. Adverse events: TACO, TRALI, Sepsis, MOF	
8. Thromboembolic complications	
9. All cause mortality at 4, 6, 24 h and up to 90 days	
Feasibility outcome measures:	
1. Time to randomisation	
2. FC and Cryo wastage	
3. Proportion of patients with blood sampling at all prespecified time points	
4. Number of missed patients (eligible but not enrolled)	
5. Randomisation errors	
6. Protocol violations	

*APTT* Activated Partial Thromboplastin Time, *Cryo* cryoprecipitate *EXTEM CT* clotting time, FBC full blood count, *FC* fibrinogen concentrate, *FFP* fresh frozen plasma, *FibC* Clauss Fibrinogen, *ICU* intensive care unit, *INR* International Normalised ratio, *LOS* length of stay*, MOF* Multiple organ failure, *PCC* Prothrombin complex concentrate, PRBC packed red blood cells, *ROTEM*® thromboelastometry, *TACO* transfusion-associated circulatory overload, *TRALI* transfusion-related acute lung injury

### Blood sampling

As previously stated the requirement for fibrinogen supplementation will be guided by FIBTEM A5 (ROTEM® Sigma) analysis. A number of other blood tests to assess haemostasis will be performed at predefined time points from admission to day 7 (Fig. 2). These include: ROTEM® Sigma and Delta (FIBTEM, EXTEM, INTEM, HEPTEM), Multiplate® (ADP, ASP, TRAP), TEG 6S (CK, CKH, CRT, CFF), haemoglobin, platelet count, International Normalised Ratio (INR), Activated Partial Thromboplastin Time (APTT), Clauss Fibrinogen. Arterial blood gas analysis (pH, lactate, base deficit, HCO_3_^−^) will be performed on admission and as clinically indicated. Likewise, blood chemistry results assessing organ function will be performed at the discretion of the treating clinical staff.

### Sample size

**Fig. 3:**
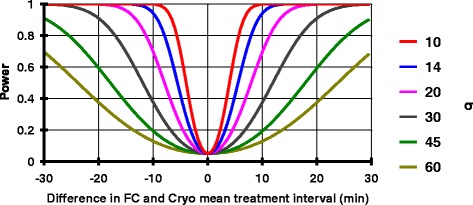
Sample size calculation

The total pilot trial sample size of 100 patients (allocated 1:1 to FC or Cryo) was set based on estimated recruitment rates across four sites and on available budget. An anticipated 70% of participants will be male, consistent with the usually observed sex ratio in severe trauma patients. Using local data combined with the published interquartile range of time intervals in a recent trial of early Cryo administration for major traumatic haemorrhage, an approximate standard deviation (SD) was derived of 14 min for each treatment arm primary outcome. Assuming this SD and normally distributed time intervals, a trial with 100 evaluable patients, equally divided between two groups, would have approximately 80% power to detect a difference of ± 8 min between mean FC and Cryo treatment intervals, at a type 1 (alpha) error of 5%. With a fixed (*n* = 100) total sample size, the SD of time intervals actually observed will have a marked influence on trial power, as summarised in Fig. 3. For example, an increase in each group SD from 14 min to 30 min would increase the detectable difference between mean responses of experimental and control subjects from 8 min to 17 min. Additionally, 100 patients recruited across four sites will enable accurate assessment of the feasibility outcome measures which are an important component of this pilot trial.

### Statistical analysis

Data analyses will be performed with a coded binary treatment indicator to preserve blinding to treatment allocation until the trial report is finalised by the writing committee. The trial primary and main secondary outcomes will be reported using intention-to-treat analyses based on all randomly assigned patients, except any who withdraw consent for use of all trial data.

Baseline variables will be summarised using descriptive statistics. The trial primary outcome and other time-to-event outcomes will be displayed as Kaplan-Meier curves and compared between treatments with Cox proportional hazards regression including, as covariates, the trial site stratification factor and patient age and sex. Sensitivity analyses will be performed using unadjusted Cox models which include a binary indicator of treatment as the only covariate, and also using multivariable Cox models including any other baseline covariates exhibiting substantial imbalance between randomisation arms. Lack of proportionality in any Cox model on testing will result in that outcome being assessed using a log-rank test and summarised using the Kaplan-Meier estimated median time to event [34]. Other secondary analyses, including assessment of the differential proportion of thromboembolic complications and mortality at specified time points, will be compared using either chi-square or Fisher exact tests, and also using logistic regression adjusting for the same covariates used in the Cox models. Results will be summarised using frequency tables, and risk ratios or odds ratios as appropriate, with 95% confidence intervals. Approximately normally distributed continuous variables (either untransformed or following log or other normalising transformation) will be compared using *t* tests, or otherwise compared using nonparametric methods (Mann-Whitney).

The differential trajectory of fibrinogen levels over time according to intervention will be assessed with fibrinogen measurements at predefined time points for each patient. Initial exploratory analyses will be conducted using a ‘summary measures’ approach [35], comparing maximum and minimum fibrinogen levels within individual patients during the acute resuscitation phase and also the maximum fibrinogen in the postresuscitation phase. Subsequently, univariable and adjusted multivariable linear regression analyses will be used to assess the differential effects on circulating fibrinogen levels of treatment allocation using a generalised estimating equation (GEE) approach with robust error estimates to account for the within-subject correlation of fibrinogen levels over time [36].

### Data and Safety Monitoring Committee and interim safety analysis

An independent Data and Safety Monitoring Committee (DSMC) will conduct two equally spaced interim analyses, at one third (*n* = 33) and two thirds (*n* = 67) of the time into trial recruitment, to assess the differential proportions of all-cause hospital mortality (censored at 30 days for practical reasons) observed in intervention (FC) compared with control (Cryo) patients.

**Fig. 4:**
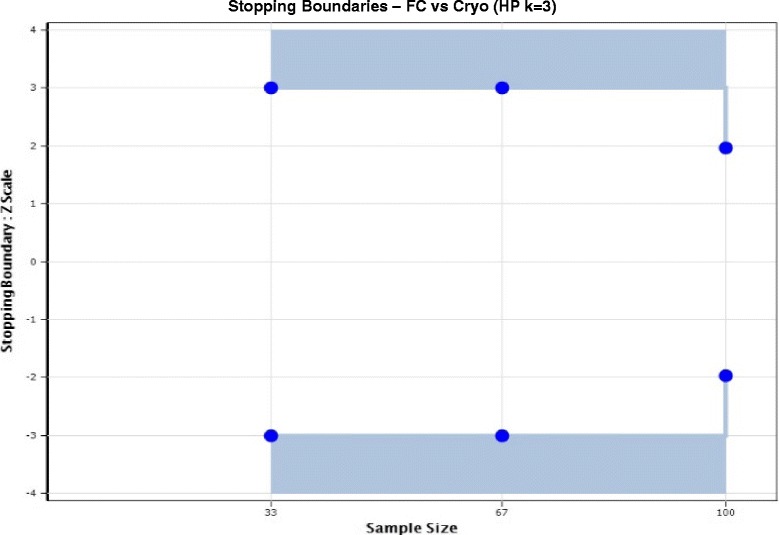
Interim analysis

These interim analyses will follow a group sequential plan (Fig. 4) employing symmetrical, two-sided, Haybittle-Peto, three-standard deviation thresholds to evaluate a standardised test statistic (|*Z*_*k*_| ≥ 3) calculated from a normal approximation to the discrete binomial difference observed between treatment and control groups in mortality. Assuming no early stopping, *k* = 3 planned analyses will occur, with all-cause mortality assessed at *n* = 33, 67 and 100 patients (Fig. 4). Accounting for the type I error spent in these two interim mortality analyses, and in the absence of early stopping, a precise analysis of the trial primary outcome at full recruitment could be conducted at *p* < 0.048 (|*Z*_*3*_| > 1.975) rather than the conventional statistical significance level (*p* < 0.05; |*Z*_*1*_| > 1.96). However, in accord with common practice with a small number of interim analyses and symmetrical two-sided, three-standard deviation, Haybittle-Peto thresholds, this trial’s primary analysis will ignore the very small error spent conducting two interim analyses, and evaluate the final primary trial outcome at the conventional two-sided level of 0.05. The DSMC may conduct additional interim analyses of mortality or other outcomes at the committee’s discretion with adjustment to the final level of significance (*p* value) if required.

## Discussion

There is increasing recognition and substantial evidence to support the importance of fibrinogen in effective clot formation in traumatic haemorrhage. Hypofibrinogenaemia is associated with reduced clot strength, increased transfusion requirements and worse outcomes [9, 12]. However, the optimal timing, method and dosing of fibrinogen supplementation remains under debate.

The PROPPR trial has shown that early, high fixed-ratio transfusion of blood products in traumatic haemorrhage is beneficial in terms of achieving haemostasis and reducing early death from exsanguination [31]. However, the early and empiric delivery of specific fibrinogen-containing products is not standard in the majority of fixed-ratio MHP. A number of studies have reported that the fixed-ratio MHP is ineffective in maintaining adequate fibrinogen levels [24, 37]. Additionally there can be significant time delays to Cryo transfusion and a large proportion of patients do not receive Cryo as part of a fixed-ratio MHP [38, 39]. The CRYOSTAT trial has shown that it is feasible to utilise Cryo empirically as part of a fixed-ratio MHP; however, the median time to transfusion was still 60 min [28].

One of the major advantages in using FC is the rapid time to delivery of fibrinogen supplementation. FC can be stored in the trauma bay, rapidly reconstituted and administered. The recently published FiiRST trial that reports early FC use is feasible and increases plasma fibrinogen levels during traumatic haemorrhage [40]. The recently completed, but not yet published, E-FIT trial (ISRCTN67540073) investigated empiric fixed-dose FC delivery in severe trauma in addition to standard MHPs, with time to delivery of FC as the primary endpoint.

There is growing evidence to support the use of VHA to rapidly guide targeted factor replacement in traumatic haemorrhage but data from RCTs is limited [41, 42]. A recent, single-centre RCT has reported significant reductions in blood product transfusion and improved survival with a VHA-guided MHP [43]. Whilst the evidence of a positive impact on mortality is equivocal, it is clear that VHA can be used to rapidly and reliably identify coagulopathic trauma patients [44, 45]. The FIBTEM assay can be utilised as a marker of TIC, predict massive transfusion and it correlates well with standard laboratory measures of fibrinogen concentration [11, 46, 47]. The present study will utilise the FIBTEM assay to identify patients with hypofibrinogenaemia and subsequently guide dosing of FC and Cryo. The FIBTEM A5 will be used to permit rapid intervention [48]. Each of the trauma centres involved in the trial have varying degrees of experience with VHA use in severe trauma [49]. This trial will utilise the ROTEM Sigma device (an automated cartridge-based system) to evaluate FIBTEM A5 and guide subsequent fibrinogen replacement. The location of the device either, at the point of care (POC) or in the laboratory environment, will depend on local logistical considerations and will be decided by each individual centre. Other blood product components will be administered as per each individual trauma centre’s standard MHP. Specifically, the use of tranexamic acid (TXA) as an intervention for hyperfibrinolysis will be as per standard practice at each individual trauma centre. The majority of Australian trauma centres are currently participating in the randomised controlled PATCH trial, investigating the prehospital use of TXA in severely injured trauma patients likely to have TIC [50]. Co-enrolment in PATCH and FEISTY has been agreed by both trial teams.

Dosing of fibrinogen utilising either Cryo or FC is controversial with widespread variability in recommendations [6]. Current European Trauma Guidelines propose FC 3–4 g or Cryo 50 mg/kg in severe traumatic haemorrhage [16]. Collins et al. describe a theoretical model of fibrinogen dosing using plasma, Cryo and FC [51]. Although not designed for clinical use it demonstrates the different volumes of each product required to achieve a desired increment in plasma fibrinogen. The recently completed FIinTIC trial may provide further evidence; this study randomised prehospital trauma patients to receive FC or placebo, with admission fibrinogen levels as the primary endpoint [52]. The currently recruiting PRooF-ITH trial is randomising patients with traumatic haemorrhage to receiving 60–70 mg/kg FC or placebo on arrival in the trauma unit, with the primary endpoint being change in thromboelastography (TEG) Functional Fibrinogen Amplitude at 15 min after intervention [53]. Cryoprecipitate dosing is made more complex by the varying fibrinogen content in each unit and the variable dose response as measured by plasma fibrinogen levels [27, 54, 55]. Published and institutional data suggest that 1 g of fibrinogen (FC or Cryo) will result in an increment of 1–2 mm in the FIBTEM assay or 0.25 g/L using Clauss Fibrinogen [47, 56–58]. The dosing schedule (FC and Cryo) for our trial has been designed in conjunction with our collaborators from the Australian Red Cross Blood Service to provide equitable dosing in both the FC and Cryo arms. To achieve a 1-g dose of fibrinogen supplementation 3–4 units of whole blood Cryo will be required. The Cryo dosing schedule will allow for approximately 250–300 mg of fibrinogen per unit of whole blood Cryo and will also allow blood banks to dispatch apheresis Cryo when available.

A significant concern regarding the use of FC is the potential for subsequent thromboembolic complications. However, data from preclinical animal studies, a recently published pharmacovigilance study, large observational studies and a systematic review would suggest that there is no increased risk of thromboembolic complications associated with FC transfusion [59–62]. Additionally, there is no evidence that even with large doses of fibrinogen supplementation, plasma fibrinogen levels exceed what would be expected subsequent to severe trauma [28, 63].

A number of trials investigating FC use in non-traumatic haemorrhage have recently been published. The FIB-PPH trial reported no benefit to empiric FC (versus placebo) in patients with clinically significant postpartum haemorrhage; however, the mean plasma fibrinogen levels were >4 g/L [64]. The REPLACE trial investigating bleeding cardiac surgical patients also reported no benefit to FC [65]. The ZEPLAST trial, also in cardiac surgery, reported reduced bleeding and transfusion requirements in patients treated with a fixed dose of 6 g FC; however, subsequent analysis suggested that a smaller dose would likely have yielded the same results [66, 67]. These trials suggest that, based on clinical indications, it is difficult to predict those patients who will benefit from supplemental fibrinogen and there is no benefit in treating those who are not hypofibrinogenaemic. Therefore, it would seem logical to treat patients in whom hypofibrinogenaemia is contributing to ongoing haemorrhage and to dose-replace based on degree of hypofibrinogenaemia.

There is an expanding observational evidence base to support the use of FC in severe trauma, with recent academic literature reporting: increased clot strength, reduction in blood loss, reduced transfusion and mortality [14, 61, 68]. A recently published systematic review found limited evidence comparing FC to Cryo and concluded that it was not possible to recommend one product over another [69]. It is imperative that robust and clinically relevant trials are performed to investigate fibrinogen replacement strategies in severe trauma before widespread practice changes are implemented without a firm evidence base [70, 71].

The large doses of Cryo utilised in traumatic haemorrhage can put strain on local blood banks in supplying requested units in a timely manner. Additionally, the size of Australia introduces logistic challenges to the maintenance of adequate Cryo stocks to individual hospital blood banks. The use of a factor concentrate with a long shelf life and that is easy to use is an attractive option but at this time is not supported by good-quality evidence.

To our knowledge, FEISTY is the first RCT comparing FC to Cryo for fibrinogen supplementation in severe traumatic haemorrhage. The trial expands on the currently utilised VHA-guided approach at the study sites, with fibrinogen supplementation and dosing guided by FIBTEM analysis [72]. In addition to assessing the differential time to effective fibrinogen supplementation, this trial will provide information on appropriate dosing of fibrinogen in traumatic haemorrhage. The results of this pilot trial in conjunction with other ongoing trials will be used to design a larger trial powered to address patient-centred outcomes such as blood product transfusion requirements and mortality.

### Trial status

The trial commenced recruitment on 23 December 2016 with an estimated 15-month enrolment period. To date, 40/100 patients have been enrolled.

## Additional file

Additional file 1: FEISTY SPIRIT Checklist (DOC 66 kb).

## Abbreviations

A5: Amplitude of clot firmness 5 min after CT
ABC Score: Assessment of Blood Consumption Score
Cryo: Cryoprecipitate
CT: Clotting time
DCR: Damage Control Resuscitation
DSMC: Data Safety Monitoring Committee
ED: Emergency Department
FC: Fibrinogen concentrate
FFP: Fresh frozen plasma
FibC: Clauss Fibrinogen
GCUH: Gold Coast University Hospital
Hb: Haemoglobin
MCF: Maximum clot firmness
MHP: Major haemorrhage protocol
OR: Operating room
POC: Point of care
PRBC: Packed red blood cells
PT: Prothrombin Time
rPT: Rapid Prothrombin Time
SLT: Standard laboratory test
TIC: Trauma-induced coagulopathy
VHA: Viscoelastic haemostatic assay
