# Supplementary material for: Fibrinogen Early In Severe Trauma studY (FEISTY): study protocol for a randomised controlled trial
Source: Trials. 2017 May 26;18:241. doi: 10.1186/s13063-017-1980-x (PMC5446750; doi:10.1186/s13063-017-1980-x)
Supplement: Additional file 1: — FEISTY SPIRIT Checklist (DOC 66 kb). [file 13063_2017_1980_MOESM1_ESM.doc]

FEISTY SPIRIT Checklist

| Scientific Title | **Fibrinogen Concentrate vs. Cryoprecipitate in Traumatic Haemorrhage: A Pilot Multi-centre Randomised Controlled Trial** |
| --- | --- |
| Short Title | **F**ibrionogen **E**arly **I**n **S**evere **T**rauma stud**Y** (**FEISTY**) |
| Health Condition | Traumatic Haemorrhage |
| Ethics | HREC/16/QGC/128 (Gold Coast Health) |
| Trial Registration | ClinicalTrials.gov NCT02745041 |
| Protocol Version | Version 2, September 2016 |
| Funding | **Grants:**  National Blood Authority Australia  Emergency Medicine Foundation  Gold Coast Health  **Industry:**  CSL Behring – Supply of Fibrinogen Concentrate  TEM International – Supply of devices and reagents |
| Primary Sponsor | Investigator Initiated and Driven Study  Dr James Winearls, Gold Coast University Hospital  1 Hospital Boulevard, Southport, QLD, Australia, 4215  Email: james.winearls@health.qld.gov.au |
| Health Problem | Traumatic Haemorrhage |
| Background | Major haemorrhage in the setting of severe trauma is associated with significant morbidity and mortality. Haemorrhage is compounded by Trauma Induced Coagulopathy of which hypo/dysfibrinogenaemia plays a significant role. There is good evidence to suggest that hypofibrinogenaemia in trauma is associated with worse outcomes and it is postulated that early replacement may reduce haemorrhage and improve outcome. This study will assess the clinical and laboratory effects of targeted dose of Fibrinogen Concentrate (FC) vs. standard of care (Cryoprecipitate) in traumatic haemorrhage. It is hypothesised that it will be significantly quicker to administer FC than Cryoprecipitate. |
| Hypothesis | Fibrinogen replacement in traumatic haemorrhage can be achieved quicker with a more predictable dose response using FC compared to Cryoprecipitate. |
| Study Aims | 1. To investigate the feasibility of early Fibrinogen replacement in traumatic haemorrhage utilising either Fibrinogen Concentrate or Cryoprecipitate 2. Compare time to administration of Fibrinogen replacement between Fibrinogen Concentrate and Cryoprecipitate 3. Investigate the effects of Fibrinogen replacement (utilising either FC or Cryoprecipitate) on Fibrinogen levels during haemorrhage |
| Study Design | - Multi-centre – GCUH / RBWH / PAH / Townsville - Randomised Controlled / Un-blinded / Feasibility - Interventional |
| Setting | 4 Major Trauma Centres in QLD, Australia  Trauma Unit / Operating Theatre  Patients can be randomised in any of the above locations |
| Inclusion and Exclusion Criteria | **Inclusion Criteria:**   1. Adult affected by Trauma (>18yrs) *and* 2. Judged to have significant haemorrhage *or* 3. Predicted to require significant transfusion with ABC Score ≥ 2 or by treating clinician judgement   **Exclusion Criteria:**   1. Injury judged incompatible with survival 2. Pregnancy 3. Known objection to blood products 4. Known coagulation disorder 5. Previous fibrinogen replacement this admission 6. Pre-Trauma Centre fibrinogen replacement 7. Participation in competing study |

| Intervention | **Intervention Arm:**  Fibrinogen Replacement using Fibrinogen Concentrate as per ROTEM guided treatment algorithm [FIBTEM ≤ A5 10mm]  **Comparator Arm:**  Fibrinogen replacement using Cryoprecipitate as per ROTEM guided treatment algorithm [FIBTEM A5 ≤ 10mm]  All other aspects of the damage control approach to severe trauma remain the same in both groups |
| --- | --- |
| Fibrinogen Dosing | **Intervention Arm (FC):**  FIBTEM A5 0mm (Flat Line) 🡺 6g FC  FIBTEM A5 1 – 4 mm 🡺 5g FC  FIBTEM A5 5 – 6 mm 🡺 4g FC  FIBTEM A5 7 – 8 mm 🡺 3g FC  FIBTEM A5 9 – 10 mm 🡺 2g FC  **Comparator Arm (Cryo):**  FIBTEM A5 0mm (Flat Line) 🡺 20U Cryo  FIBTEM A5 1 – 4 mm 🡺 16U Cryo  FIBTEM A5 5 – 6 mm 🡺 14U Cryo  FIBTEM A5 7 – 8 mm 🡺 10U Cryo  FIBTEM A5 9 – 10 mm 🡺 8U Cryo |
| Primary Outcome Measures | 1. A) Time to administration of Fibrinogen Replacement from time of ROTEM analysis indicating fibrinogen supplementation is required (FC and Cryoprecipitate). First dose of FC or Cryo required.   B) Feasibility of administering FC within 30 mins of clinical scenario and ROTEM analysis suggesting Fibrinogen replacement is required.   1. Effects on Fibrinogen levels during traumatic haemorrhage as measured by Clauss Fibrinogen and FIBTEM analysis. |
| Secondary Outcome Measures | 1. Transfusion Requirements (in number of units of PRBC, FFP, FC, Cryoprecipitate, Platelets, PCC at 4, 6, 24, 48hrs) 2. Duration of bleeding episode or time until surgical control and with no further requirement of coagulation factors 3. Duration of ICU and Hospital LOS 4. Duration of Mechanical Ventilation 5. ROTEM (Sigma and Delta), Multiplate, FBC, INR, APTT, FibC analysis at pre-specified time points:  - At ED Presentation - Or recognition of significant haemorrhage - 10 mins after intervention - As clinically indicated - After every 4 Units PRBC - At ICU admission, +6hrs, +12hrs and then daily for 7 days  1. Specific evaluation of EXTEM CT (Clotting Time) during active haemorrhage and in response to Fibrinogen replacement 2. Adverse Events – TACO / TRALU / Sepsis / MOF / ARF 3. Thromboembolic Complications 4. All cause Mortality at 4, 6, 24 hours and up to 90 days |
| Feasibility Outcome Measures | 1. Time to randomisation 2. FC or Cryoprecipitate wastage 3. Proportion of patients with blood sampling at all pre-specified time points 4. Number of missed patients (eligible but not enrolled) 5. Randomisation errors 6. Protocol violations |
| Sample Size | 50 patients in each arm / 100 Patients Total |

| Randomisation | Randomisation will occur on arrival in the Trauma Unit if:   1. Adult affected by trauma 2. Judged to have significant haemorrhage 3. ABC ≥ 2 or Clinically judged to require MT   At this stage patients will be randomised into either the Fibrinogen Concentrate or Cryoprecipitate arms   - The requirement for Fibrinogen replacement is triggered by a FIBTEM A5 ≤ 10mm - Once the patient reaches this trigger then fibrinogen replacement will be carried out as per randomisation (i.e. FC or Cryo) - Patients will remain in their initial randomisation group throughout hospital admission |
| --- | --- |
| Randomisation Mechanisms | - Eligibility assessment and Randomisation performed by trained trauma staff - Computer generated randomisation - Secure Web based - 1:1 Block randomisation schedule |
| Blinding | - Un-blinded to patients and health care providers - Blinded to outcome assessors and data analysts |
| Data Collection Methods | - Secure web based electronic CRF - Data entry performed by trained research staff - Data collected from individual patient record |
| Data Collected | - Patient Demographic Data - Time of arrival in Trauma Unit - Time of randomisation - Time of intervention - Blood Products – Time + Quantity of transfusion - Laboratory / ROTEM / Multiplate data - ISS / APACHE / SOFA - Transfusion related adverse events - Clinically relevant thromboembolic events |
| Statistical Analysis | - Intention to treat analysis - Statistician blinded to intervention allocation |
| Data and Safety Monitoring | - DSMC – Statistician / Intensivist / Trauma Surgeon - SAE reported to Coordinating site within 24hrs - DSMC to review all SAE - DMSC to make recommendations to PI in event of study conduct or SAE issues - Interim safety specific analysis at 1/3 (33 patients) and 2/3 (66 patients) time points - Study coordinator will monitor trial conduct at each study site at regular intervals - Participation in CSL pharmaco-vigilance programme with respect to Fibrinogen Concentrate |
| Ethics and Governance | - HREC approval has been granted by GCHHS - Clinical governance (Site Specific Authority) procedures will be followed for all participating sites - Protocol amendments will be submitted to lead ethics site with appropriate dissemination to participating study sites - The study will be conducted in accordance with GCP |
| Consent | - Where possible informed consent will be obtained from participants - This may not be possible in the setting of severe trauma especially as the intervention is time critical - In this scenario consent will be sought from next of kin - If no next of kin available then emergency care research process will be followed allowing enrolment into the study with the agreement of a senior clinician subsequent consent will be sought as soon as possible from patient or representative |
| Confidentiality | - All patient details will be de-identified - Data will stored in a de-identified, secure electronic form |
| Dissemination | - Trial results will published in peer reviewed medical literature and/or presented at relevant scientific meetings |
| Study Status | - Recruitment anticipated December 2016 - 15 month recruitment - 6 month data analysis manuscript preparation |
